# Under-triage of older trauma patients in prehospital care: a systematic review

**DOI:** 10.1007/s41999-021-00512-5

**Published:** 2021-06-10

**Authors:** Abdullah Alshibani, Meshal Alharbi, Simon Conroy

**Affiliations:** 1 Department of Health Sciences, College of Life Sciences, University of Leicester, Leicester, LE1 7HA UK; 2 Emergency Medical Services Department, College of Applied Medical Sciences, King Saud Bin Abdulaziz University for Health Sciences, Riyadh, Saudi Arabia; 3 Department of Cardiovascular Sciences, College of Life Sciences, University of Leicester, Leicester, UK

**Keywords:** Geriatrics, Injury, Silver trauma, Triage, Paramedics, Emergency

## Abstract

**Background:**

It is argued that many older trauma patients are under-triaged in prehospital care which may adversely affect their outcomes. This systematic review aimed to assess prehospital under-triage rates for older trauma patients, the accuracy of the triage criteria, and the impact of prehospital triage decisions on outcomes.

**Methods:**

A computerised literature search using MEDLINE, Scopus, and CINHAL databases was conducted for studies published between 1966 and 2021 using a list of predetermined index terms and their associated alternatives. Studies which met the inclusion criteria were included and critiqued using the Critical Appraisal Skills Programme tool. Due to the heterogeneity of the included studies, narrative synthesis was used in this systematic review.

**Results:**

Of the 280 identified studies, 23 met the inclusion criteria. Current trauma triage guidelines have poor sensitivity to identify major trauma and the need for TC care for older adults. Although modified triage tools for this population have improved sensitivity, they showed significantly decreased specificity or were not applied to all older people. The issue of low rates of TC transport for positively triaged older patients is not well understood. Furthermore, the benefits of TC treatment for older patients remain uncertain.

**Conclusions:**

This systematic review showed that under-triage is an ongoing issue for older trauma patients in prehospital care and its impact on their outcomes is still uncertain. Further high-quality prospective research is needed to assess the accuracy of prehospital triage criteria, the factors other than the triage criteria that affect transport decisions, and the impact of under-triage on outcomes.

**Supplementary Information:**

The online version contains supplementary material available at 10.1007/s41999-021-00512-5.

## Key summary points

**Aim:**

The systematic review aimed to assess the under-triage rate for older trauma patients in prehospital care and its impact on their outcomes.

**Findings:**

Older trauma patients were significantly under-triaged in prehospital care and the benefits of triaging these patients to Tauma Centres (TCs) are still uncertain. Current triage criteria and developed geriatric-specific criteria lacked acceptable accuracy and when patients met the criteria, they had a low chance of being transported to TCs.

**Message:**

Future worldwide research is needed to assess the following aspects: (1) the accuracy of current trauma triage criteria, (2) developing more accurate triage criteria, (3) destination compliance rates for patients meeting the triage criteria, (4) factors leading to destination non-compliance and their impact on outcomes, and (5) the benefits of TC access for older trauma patients.

**Supplementary Information:**

The online version contains supplementary material available at 10.1007/s41999-021-00512-5.

## Introduction

The population of older adults is continuing to grow in numerous countries across the world. In the United Kingdom (UK), 18% of the population are now aged ≥ 65 years [1] and the number of people aged ≥ 85 years is predicted to double in the next 25 years [2]. The trend of increasing rates of population ageing is generalised to most European countries [3]. There were 90.5 million people aged ≥ 65 years living in Europe in 2019 which represented 20.3% of the total population [3]. This trend is expected to continue in Europe; peaking at 129.8 million older adults and representing 29.4% of the total population by 2050 [3]. As this population grows, more older patients will require high-quality trauma care including prehospital assessment, management, and triage decisions [4].

Current data indicates there is an increasing number of patients aged ≥ 60 years who have sustained major injuries despite apparently low impact trauma, and these now comprise half the number of patients classified as having major trauma in the UK (Injury Severity Score [ISS] > 15) [5]. Although recent studies have shown that older trauma patients with one or more comorbid health problems are at greater risk of death [6], clinical protocols and guidelines are not clear on how to optimise the treatment and management of this population [4].

Prehospital trauma triage tools are predominantly used to identify injury severity in trauma patients to determine transportation decisions [7, 8]. Any trauma triage tool should be sensitive enough to identify major trauma patients so they can benefit from access to level I/II Trauma Centres (TCs) (equivalent to Major Trauma Centres in the UK, or Major Trauma Service in Australia), whilst being specific enough to predict patients whose injuries are not classified as major trauma so they can be treated at lower or non-TCs [9]. Recent research has found that older people suffer increased under-triage in prehospital trauma care [10].

Under-triage refers to the transportation of severely injured patients to lower-level TCs or other acute care facilitates [11]. There are several factors that may affect the accuracy of prehospital trauma triage criteria and triage decisions for older adults, including altered physiological responses to trauma [12, 13], perceived insignificant injury mechanisms such as low-level falls (i.e. < 2 m) [14, 15], high comorbidity [16] and frailty rates [17, 18], and the use of multiple medications [19]. The effective triage of injured patients is the first step towards providing high-quality care and reducing mortality rates [20]. A recent piece of evidence from Sweden showed that treatment at TCs was associated with a 41% lower adjusted 30-day mortality (Odds Ratio [OR] 0.59, 95% Confidence Interval [CI] 0.50–0.70, *P* < 0.0001) compared to non-TCs for all trauma patients (i.e., ISS ≥ 1) [21]. Evidence from the United States of America (USA) also showed that TC treatment, compared to non-TC treatment, was associated with a significantly lower in-hospital mortality rate (7.6% vs. 9.5%, Relative Risk [RR] 0.80, 95% CI 0.66–0.98) and one-year mortality rate (10.4% vs. 13.8%, RR 0.75, 95% CI 0.60–0.95) [22]. Therefore, increased under-triage for older trauma patients is considered to increase their mortality rates [10]. This systematic review, therefore, aimed to assess recent evidence for prehospital under-triage rates of older trauma patients, the accuracy of current and developed prehospital trauma triage criteria for this population, and the impact of prehospital trauma triage on their outcomes.

## Methods

To effectively answer the problem of the review, an appropriate question was established using the Population, Intervention, Comparison, and Outcome (PICO) model [23]: are older trauma patients under-triaged in prehospital care? (P: older adults, I: trauma triage, C: younger adults, and O: rate of under-triage).

A literature search was undertaken using the following databases: MEDLINE, Scopus, and CINHAL. A list of index terms and associated alternatives were generated prior to performing this electronic search to identify the most relevant literature. The index terms were: ‘older adult*’, ‘trauma’, ‘under-triage’, and ‘prehospital’. The alternatives that were applied in this search included the following: ‘advanced age’, ‘elderly’, ‘geriatric*’, ‘injur*’, ‘triag*’, ‘paramedic*’, ‘emergency medical service*’, ‘EMS’, and ‘ambulance’. In addition to searching the selected databases, the reference lists of the included studies were reviewed to identify further possible studies for inclusion in this review. This systematic review was performed according to the Preferred Reporting Items for Systematic Reviews and Meta-Analyses (PRISMA) guidelines [24].

**Fig. 1:**
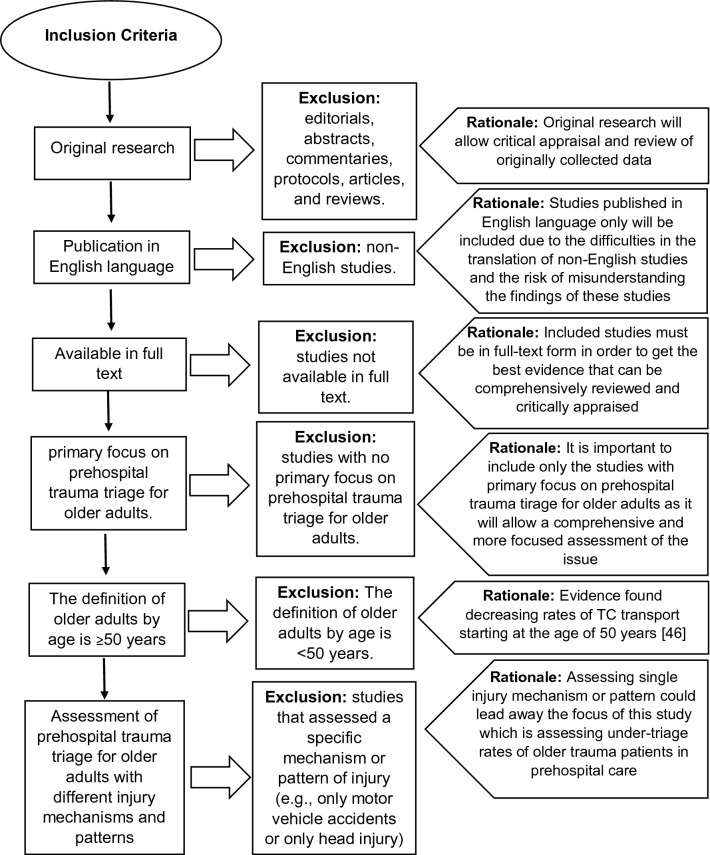
Inclusion and exclusion criteria

Clear inclusion/exclusion criteria were discussed and determined by the authors prior to search the literature (Fig. 1). No specific time limits were employed in this search as any study met the criteria and published prior to January 7, 2021, which is the date of the last database search, was included in the review.

The eligibility assessment, data extraction, and critical appraisal were performed independently by two reviewers (first and second authors). The process of critical appraisal was performed using the Critical Appraisal Skills Programme (CASP) for quantitative cohort studies [25]. The rates of under-triage among older trauma patients and their impact on the outcomes were the primary outcome measures. Under-triage, in general, is defined as transporting patients with major trauma (ISS > 15) whether they met the prehospital trauma triage or not to lower-level or non-TCs. We made sure to define under-triage in the included studies as it is sometimes defined by triage criteria, initial destination, or final destination. The sensitivity and specificity of any triage criteria could differ from one to another based on what they are sensitive or specific for, which may include, for example, higher injury severity, need for TC care, or adverse outcomes. This was considered and made clear in the results section where needed. Each study was read, reread, critically appraised, and assessed for the risk of bias including the assessment of selective reporting bias independently by two reviewers. The main findings of each study were collated, assessed for further analysis, and are interpreted in the discussion. A third reviewer was assigned in case of disagreement about inclusion/exclusion criteria or quality assessment of the included studies in the systematic review. Due to the heterogeneity of the included studies, a thematic interpretation was applied [26]. Sub-themes were generated from each included study first which then were merged into more representative themes.

## Results

**Fig. 2:**
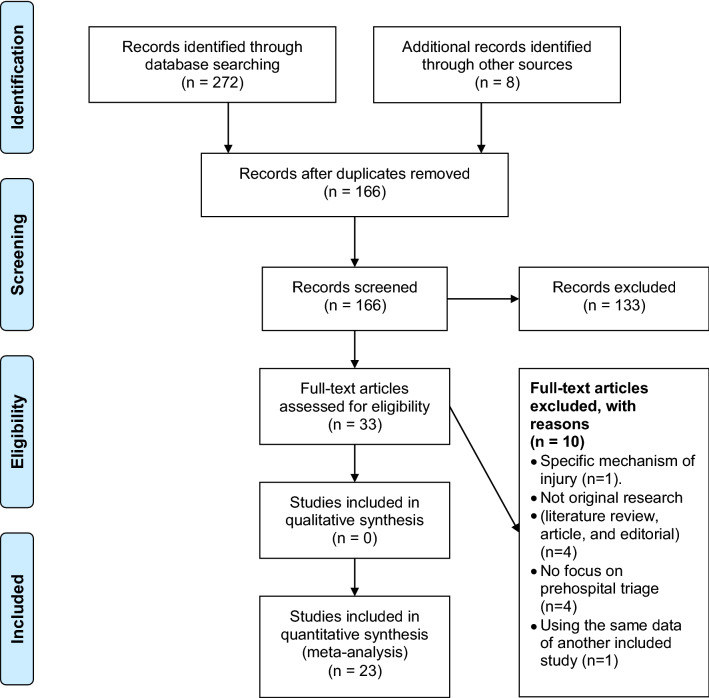
PRISMA flow diagram of the systematic review

Of the 280 screened articles, 33 abstracts were found relevant to the initial inclusion in this review (Fig. 2). After obtaining and reviewing full-text articles, 10 studies were excluded as they were not original research (literature review, articles, and editorial) [27–30], not focused in evaluating prehospital triage [10, 31–33], assessed a specific injury mechanism [34], or used the same data of another included study [35]. The quality assessment of the remaining 23 articles was performed independently by two reviewers. There were no disagreements between the two reviewers.

Six of the 23 included studies did not mention any information about ethical approval [36–41]. Two studies may have a risk of selection bias; one study included all patients coming to the emergency department whether by ambulance or private vehicle which could adversely affect the assessment of the accuracy of prehospital triage decisions [42], and one study restricted its sample to those who had pre-injury information [43]. Overall, all the included articles were of moderate quality.

Most of the included studies were conducted in the USA (*n* = 21) [36, 38–57]. Eleven studies were published in the last five years [41, 43–45, 47, 50, 52, 55–58], four of which were published in 2020 [41, 55–57]. All studies were retrospective cohort studies and only one study additionally included a survey (Table 1). Furthermore, data were collected retrospectively for different periods of time ranging from six months to up to 13 years (Table 1). The included studies had different definitions of the population of older adults by age (Table 1). Moreover, some studies included younger adults and children, comparing their prehospital trauma triage decisions with those of older adults (Table 1).

**Table 1:** Characteristics of the included studies

Characteristics		Studies	*N*	%
Year	2016–2020c	[41, 43–45, 47, 50, 52, 55–58]	11	48%
	2011–2015	[36–38, 40, 42, 48, 51, 53, 54]	9	39%
	2006–2010	[46, 49]	2	9%
	Prior to 2000	[39]	1	4%
Country	United States	[36, 38–57]	21	91%
	Australia	[37, 58]	2	9%
Design	Retrospective design	[36–45, 47–58]	22	96%
	Retrospective design + Surveys	[46]	1	4%
Data collection period	6 months	[39]	1	4%
	1 year	[38, 41–43]	4	17%
	14 months	[50]	1	4%
	2 years	[53]	1	4%
	3 years	[36, 44, 51, 52]	4	17%
	4 years	[58]	1	4%
	5 years	[37, 40, 49]	3	13%
	6 years	[45, 48, 54, 57]	4	17%
	10 years	[46, 47, 55]	3	13%
	13 years	[56]	1	4%
Sample	Only older adults	[40–45, 50, 52, 53, 55–57]	12	52%
	All Adult population	[36–38, 47–49, 54, 58]	8	35%
	Paediatric and All Adult populations	[39, 51]	2	9%
	All trauma patients + healthcare providers	[46]	1	4%
Definition of older adults by age	≥ 50 years	[50]	1	4%
	≥ 55 years	[38, 39, 41, 42, 47, 53, 55]	7	30%
	> 55 years	[37, 51]	2	9%
	≥ 65 years	[40, 43, 46, 52, 57, 58]	6	26%
	> 65 years	[36, 49, 56]	3	13%
	≥ 70 years	[44, 45, 48, 54]	4	17%

A thematic approach was applied when analysing the literature as this was appropriate given the methods employed in the articles that were reviewed [26]. Upon completion of full-text reading and critical analysis, seventeen initial sub-themes were identified and developed based on their prominence and relevance to the review. Interlinked subthemes were then combined into five overarching and representative themes (Table 2). The themes within each included study are presented in Supplement 1.

**Table 2:** Themes and inter-linked subthemes

Themes	Inter-linked Subthemes
1. Under-triage rates	– Rates of initial under-triage – Under-triage in inter-hospital transfers – Under-triage of helicopter transportations – Mode of transportation
2. Clinical effectiveness of trauma triage criteria	– Overall accuracy rates of triage criteria – Physiological factors – Comorbidities – Injury-related factors – Distance
3. Developing specific trauma triage criteria	– Modifying current trauma triage criteria – Developing specific trauma triage criteria for older adults
4. Trauma triage destination compliance	– Destination compliance rates – Patient or relative choice – Socioeconomic factors – Paramedic-related factors
5. Trauma triage and outcomes	– Hospital-related outcomes – Patient outcomes

### Under-triage rates

In the USA, nationwide research has found that older trauma patients are significantly under-triaged in prehospital care [42] (Table 3). This finding is similar to the results of other studies conducted at both regional and state-wide level in the USA [38–40, 43, 46–48, 50–53, 55–57], and Australia [37, 58] (Table 3). Under-triage is still an ongoing issue for older trauma patients for over 25 years now, however, most research work looking into this issue was done in the last decade (Table 3).

**Table 3:** Under-triage rates in the included studies

Study	Main findings
Brown et al. (2019) [58]	The majority of injured younger patients were transported to a TC (*n* = 578, 55%) whereas the majority of injured older patients were transported to a non-TC (*n* = 232 [40%], P < 0.001) The odds of transporting older trauma patients to TCs in Australia decreased with age as those who aged 65 to 74 years had a 48% reduction in the rate of TC transport (adjusted OR 0.52, 95% CI 0.35–0.78) compared to 63% reduction for others aged ≥ 85 years (adjusted OR 0.37, 95% CI 0.24–0.55) Overall, a fall from standing resulted in more than 53% reduced odds of TC transport (adjusted OR 0.47, 95% CI 0.33–0.67) Positive predictors of TC transport included motor vehicle crash (adjusted OR 2.5, 95% CI 1.6–4.0) and male gender (adjusted OR 1.4, 95% CI 1.1–1.8)
Chang et al. (2008) [46]	The rate of under-triage among older trauma patients was 50% compared to 18% for younger trauma patients (P < 001) Older trauma patients who aged ≥ 65 years had a 52% reduced chance to be transported to TCs (OR, 0.48; 95% CI 0.30–0.76) after controlling of possible confounding factors (year, sex, physiology, injury, or mechanism criteria, transport reasons, prehospital care provider training level, presence or absence of 18 specific injuries, and jurisdictional region)
Cox et al. (2014) [37]	In a univariate logistic analysis, with each increase of age by one year, the chance of being transported to TCs decreased by 2 percent (OR 0.982, 95% CI 0.982–0.983) The unadjusted odds of transporting injured older adults to a TC was 57% lower (OR 0.431; 95% CI 0.416, 0.446) than for injured younger adults
Davis et al. (2012) [38]	For injured patients who aged 15 to 54 years, 83% of them were positively triaged by Florida Trauma Triage Algorithm (FTTA) and 86% had ISS > 15 (OR 2.88, 95% CI 2.44–3.41). The OR for patients with ISS > 15 was 6.53 (95% CI 4.07–10.47) In comparison, injured patients who aged ≥ 55 years, 59% of them who were positively triaged by FTTA and 64% had ISS > 15 (OR 1.03, 95% CI 0.93–1.15). (OR 1.67, 95% CI 1.08–2.58). The OR of the triaging effect for patients aged ≥ 55 years with ISS > 15 was slightly increased (OR 1.67, 95% CI 1.08–2.58) compared to those with lower injury severity (i.e., ISS 0–15) (OR 1.00, 95% CI 0.89–1.12)
Garwe et al. (2017) [47]	Older injured adults had a higher chance of being transferred to non-TCs in comparison with younger trauma patients (53% vs. 34%, p < 0.05) Older injured patients also had a less chance to be transported by HEMS (14.6% vs. 20%, p < 0.05) After controlling for confounding factors and distance measures, the study showed that older trauma patients had a significantly less chance of being transported to and treated at TCs (OR = 0.54, 95% CI 0.52–0.56), whether they were initially transported by ambulance from the scene (OR = 0.47, 95% CI 0.44–0.50) or through inter-facility transfers from non-TCs (OR = 0.63, 95%CI 0.59–0.68)
Garwe et al. (2020) [55]	The results of this study showed that 57% of older trauma patients were treated at non-TCs compared to 43% at TCs Patients treated at TCs were younger, predominantly (P < 0.05) male, had traffic-related or penetrating injuries, more likely to be transported by ambulance from the injury scene, and injured to place close from tertiary or level III TCs Patients aged ≥ 65 years had a disproportionately higher rate of treatment at non-TCs than that at TCs (82% vs 64%) and the majority of the injuries (82%) were fall-related
Horst et al. (2020) [56]	The median under-triage rate for older trauma patients was 50.5% (Inter-quartile Range [IQR], 38.2–60.1%)
Ichwan et al. (2015) [48]	When the outcome is determined as ISS > 15, the current triage guidelines showed a high sensitivity for younger adults (87%; 95% CI 86%-87%), but a significantly decreased sensitivity for older adults (61%; 95% CI 60%-62%)
Kodadek et al. (2015) [42]	There was a reduction trend in transporting patients to lower TCs with increased NISS as 76%.2 of patients with NISS < 9 were transported to lower or non-TCs which then decreased to 66.2% with NISS between 9 to 15, and 44.8% with NISS between 16 to 24 However, for patients who had NISS ≥ 25, 54.1% of them were treated at lower or non-TCs Even when older trauma patients living in rural areas were excluded, the rate for under-triage was still high (55.8%) For older trauma patients, most injuries treated at lower or non-TCs as well as those at higher level TCs were resulted from falls (71.3% and 59.3%, respectively). However, 16.5% of patients with injuries resulted from motor vehicle accidents were treated at higher level TCs compared to only 5% who were treated at lower or non-TCs (P < 0.001)
Meyers et al. (2019) [50]	The proportion of geriatric patients meeting physiological criteria of the Trauma Triage Destination Plans (TTDP) who were transported to TCs was 24.4% pre-TTDP and 24.4% post-TTDP Few patients bypassed a closer hospital to a TC (pre-TTDP, 12.6% [*n* = 250; 95% CI 11.1–14.1]; post-TTDP, 11.9% [*n* = 282; 95% CI 10.6–13.2] Even when trauma was within 60 min from a TC, still few patients bypassed to a TC (pre TTDP, 17.9% [*n* = 220; 95% CI 15.8–20.1]; post-TTDP, 16.7% [*n* = 248; 95% CI 14.8–18.6]) Although no difference was found between the pre- and post-time interval when the trauma occurred more than 60 min from a TC (pre-TTDP, 4.0% [*n* = 30; 95% CI 2.6–5.3]; post-TTDP, 3.9% [*n* = 34; 95% CI 2.6–5.1]), the rate for transporting patients to a TC declined dramatically when the 60 min threshold was crossed at both intervals (pre-TTDP 13.9% decrease; post-TTDP 12.8% decrease) Increasing age was associated with decreased rates of TC transport (*P* < 0.05). The rates of TC transport for patients aged 50–59 years pre- and post-TTDP were 30.5% (*n* = 185; 95% CI 26.9–34.2) and 30.7% (*n* = 213; 95% CI 24.5–36.9), compared to patients > 80 years pre- and post-TTDP 19.9% (*n* = 157; 17.09–22.65) and 19.5% (*n* = 185; 17.0–22.0; *P* < 0.05) Almost 3% of the entire study population of older trauma patients were transported through HEMS although 31% of them met the trauma triage criterion (≥ 60 min from the nearest TC)
Nakamura et al. (2012) [51]	Under-triage rate was relatively constant, but then progressively increased after the age of ≥ 60 years and reached a rate of 58% to 62.2% among older patients aged > 90 years
Newgard et al. (2016) [52]	The sensitivity and specificity of current trauma triage criteria: For ISS > 15: sensitivity 75.9%, 95% CI 72.3–79.2%; specificity 77.8%, 95% CI 77.1–78.5% (Area Under the Curve (AUC) 0.77 [0.75–0.79]), (146 out of 605 patients were under-triaged) For serious traumatic brain injury: sensitivity 64.5%, 95% CI 60.8–68.2%; specificity 77.4%, 95% CI 76.6–78.1% (AUC 0.71 [0.69–0.73]), (225 out of 634 patients were under-triaged) For serious chest injury: sensitivity 57.2%, 95% CI 52.6–61.7%; specificity 76.5%, 95% CI 75.8–77.3% (AUC 0.67 [0.65–0.69]), (194 out of 453 patients were under-triaged) For serious abdominal-pelvic injury: sensitivity 38.6%, 95% CI 32.4–44.9%; specificity 75.6%, 95% CI 74.9–76.4% (AUC 0.57 [0.54–0.60]), (143 out of 233 patients were under-triaged)
Newgard, et al. (2019) [43]	The study showed the poor sensitivity of the current trauma triage criteria as they identified only 117 out of 320 patients who had ISS > 15 and those whose injuries required a major non-orthopedic surgery The sensitivity of current trauma triage criteria for older trauma patients was 36.6% (95% CI 31.2–42.0%) and the specificity was 90.1% (95% CI 89.2%–91.0%) Out of the 5021 injured older adults who are included in this study, only 803 (16%) were initially transported to higher-level TC Of the 583 patients who met the current triage criteria, 222 (38.1%) were transported to higher-level TC When measuring triage based on the hospital destination, 114 patients of 320 who had an ISS > 15 or those who required non-orthopedic surgery were initially transported to a Level I/II TC (sensitivity 35.6%; 95% CI 30.1%–41.1%) Patients who did not have serious injuries or require specialised operations (*n* = 4701), 689 of them were transported to TCs (specificity, 85.3% [95% CI 84.3%–86.3%]) Of patients who were seriously injured but transported to non-TCs (*n* = 206), 51 (24.8%) of them were ultimately transported to higher-level TCs. This resulted in a sensitivity of 50.8% (95% CI 45.0%– 56.6%) and specificity of 84.5% (95% CI 83.5%–85.6%) when the triage is defined by final destination to higher-level TCs
Phillips et al. (1996) [39]	Among injured older adults aged ≥ 55 years, the sensitivity was 29% with an under-triage rate of 71% while the specificity of the triage criteria was 92.6% with an over-triage of 7.4% Among injured younger adults aged 15–54 years, the sensitivity was 64% with an under-triage rate of 36% while the specificity of the triage criteria was 88.7% with an over-triage of 11.3%. Although the rate of under-triage wasbelow the target level of not more than 5%, the study argued that this rate is comparable to the results of other studies The rate of under-triage among older trauma patients increased with age; reaching a rate of 81.9% for those aged ≥ 85 years According to the mechanism of injury, the triage criteria were highly sensitive to gunshot wounds (under-triage rate of 5%) and significantly less sensitive to falls (under-triage rate of 94.3%)
Pracht et al. (2011) [40]	The rates of TC treatment decreased with age as 50.31% of patients aged 65 to 74 years were treated at TCs compared to 35.85% among patients aged 75 years to 84 years and 27.19% among patients aged ≥ 85 years
Staudenmayer et al. (2013) [53]	Older trauma patients were significantly under-triaged in pre-hospital care (32.8% of patients with ISS > 15 were under-triaged) When under-triage is defined to include all patients transported to non-TCs either they had ISS > 15 or a procedure including interventional radiology or non-orthopedic surgery, the rate of under-triage increased to 44%
Uribe-Leitz et al. (2020) [57]	For trauma patients aged ≥ 65 years old, 26.5% of them were treated at TCs compared to 73.5% at non-TCs The rate of under-triage for patients with ISS > 15 was 46.3% The rate of under-triage increased with age; reaching 57% for patients aged > 80 years (OR 1.52; 95% CI 1.52–1.61)

Under-triage rates were shown to be relatively constant in trauma patients up to 60 years, then increased with age up to a rate of 58% to 62% in patients aged > 90 years [51]. Further evidence from the USA and Australia showed increased under-triage with increasing age among older trauma patients [40, 58] (Table 3).

Evidence from Australia also showed that, even though current adult prehospital trauma triage criteria are more sensitive in identifying major trauma (ISS > 15) for older adults than those described in other research studies [38–40, 42, 43, 46–53, 55–57], older trauma patients were significantly under-triaged in comparison with younger adults [37]. Even during inter-hospital transfers, older trauma patients were less likely to be transferred to higher TCs. For example, 53% of older patients presenting at non-TCs were transferred to other non-TCs compared to only 34% of younger patients [47]. In addition, 15% were transported by Helicopter Emergency Medical Services (HEMS) compared to 20% of younger trauma patients [47]. Indeed, Meyers et al*.* [50] showed that almost 3% of the entire study population of older trauma patients were transported through HEMS although 31% of them met the trauma triage criterion (≥ 60 min from the nearest TC).

Some older trauma patients were not triaged in prehospital care, which may explain the issue of increased under-triage rates in this population. The transport of patients aged ≥ 65 years by private vehicles, meaning they were not triaged in prehospital care, was associated with increased odds of non-TC treatment by 19% compared to those transported by ambulance (OR 1.19; 95% CI 1.17–1.20) [57]. Furthermore, for patients with an ISS > 15 and living > 30 miles from TCs, the private vehicle transport was associated with increased odds of under-triage by 69% (OR 1.69; 95% CI 1.62–1.76) compared to those transported by ambulance [57]. Treatment at TCs was predominantly associated with ambulance transport for older trauma patients [55].

### Clinical effectiveness of trauma triage criteria

Current trauma triage tools lack acceptable accuracy in identifying major trauma for older adults [48, 52]. Most common examples of prehospital triage tools are Field Triage Decision Scheme in the USA [59] and the Major Trauma Triage Tool in the UK [60]. Different triage tools are applied in different countries even some states, cities, or trusts within the same country have different trauma triage tools, which makes it difficult to cover all of them in this review.

A study from the USA showed that the sensitivity of current trauma triage criteria to identify major trauma (ISS > 15) and need for TC care was 61% for patients aged ≥ 70 years compared to 87% for younger patients [48]. Another study highlighted a sensitivity of almost 76% in identifying major trauma for patients aged ≥ 65 years [52]. However, research in Australia has shown that the Victorian trauma triage tool can identify major trauma among older patients as effectively as it can for younger patients with sensitivity rates of 96% [37].

There are factors that could adversely affect meeting some criteria of the triage tool for older trauma patients such as physiological criteria [47, 49, 54, 55], comorbidities [55], injury pattern and mechanism [39, 41, 53, 58] (Table 4). Distance could also affect the time to get the patient to a TC, which is used as a criterion for TC transport in some triage tools [47, 55, 57] (Table 4).

**Table 4:** Factors Affecting Accurate Prehospital Trauma Triage Decisions

Factors	Findings
***Factors related to the effectiveness of prehospital trauma triage criteria***	
Physiological variables (Systolic Blood Pressure [SBP], Heart Rate [HR], and Glasgow Coma Scale [GCS])	Systolic Blood Pressure and Heart Rate: Trauma patients aged ≥ 55 treated at TCs were more likely (*p* < 0.05) to present with hypovolemic shock (whether the SBP was < 110 mm Hg or < 90 mm Hg) than those treated at non-TCs (*p* < 0.0001) [55] Older trauma patients aged > 55 years, compared to their younger counterparts, were significantly less likely to experience shock (SBP < 90 mm Hg) (SBP, mm Hg, mean [± Standard Deviation (SD)]:144 [33] vs. 131 [29]) and also less likely to have tachycardia (HR, beat per minute, mean [± SD]: 82.7 [20] vs. 91.7 [25]) [47] This is consistent with other research findings which found decreasing rates of older patients aged > 65 years presenting with hypotension (SBP < 90 mm Hg) and tachycardia (HR > 100 beats per minute) [49]
	Glasgow Coma Scale: Trauma patients aged ≥ 55 treated at TCs were more likely (*p* < 0.05) to present with lower GCS (GCS < 9) than those treated at non-TCs (*p* < 0.0001) [55] Older trauma patients aged > 55 years, compared to their younger counterparts, had higher GCS (GCS mean [± SD]: 14.2 [2.4] vs. 13.6 [3.5]) [47] Applying the GCS ≤ 13 for trauma patients aged ≥ 70 years had much worse sensitivity; decreasing the sensitivity rate by 35%; from 85.7% (95% CI 84.1–87.2) in younger adults to 50.7% (95% CI 47.5–53.9) in older adults [54]. However, its application had higher specificity for older patients compared to younger adults; increasing the specificity rate by 8.8%; from 85% (95% CI 84.6–85.4) in younger adults to 93.8% (95% CI 93.4–94.2) in older adults [54]
Comorbidities	Patients aged ≥ 55 years treated at non-TCs were shown to have a slightly higher prevalence of comorbidities (44.7% vs 42.3%) notably the higher prevalence of preexisting cardiac-related diseases (29.1% vs 25.6%) (*p* < 0.0001) [55]
Distance	Trauma patients aged ≥ 55 years and treated at TCs were injured in places close to these centres [55] Compared to younger adults, older trauma patients suffered injuries in places that were slightly further from TTCs (47 miles vs 44 miles) [47] For trauma patients aged ≥ 65 years, distance was shown to impact prehospital trauma triage as older patients who lived > 30 miles from a TC has 37% higher odds of under-triage (OR 1.37; 95% CI 1.15–1.40) compared to those who lived within 15 miles [57]. Furthermore, for patients with ISS > 15, the association between age and under-triage was more pronounced for patients living > 30 miles from a TC; increasing the odds of under-triage by 64% for patients aged > 80 years (OR 1.64; 95% CI 1.53–1.76) [57]
Injury-related factors (injury pattern and mechanism)	Injury pattern: Patients aged ≥ 55 years who were treated in TCs had a higher incidence of serious injuries to head, chest and abdomen (i.e. Abbreviated Injury Scale [AIS] ≥ 3) (*p* < 0.0001) whereas for those treated at non-TCs, a higher incidence of serious extremity injuries was reported (i.e. long bone fractures [humerus or femur] but pelvic fractures were more likely to be treated at TCs) (*p* < 0.0001) [55]
	Mechanism of injury: Patients aged ≥ 55 years who were treated in TCs predominantly had motor vehicle accidents (*p* < 0.0001) [55]. Those treated at non-TC predominantly (82%) had fall-related injuries [55] Vehicle crashes were a predictor of TC transport for trauma patients aged ≥ 55 years (OR 3.39, CI 2.79–4.11) [41] Evidence from Australia also showed that one of the positive predictors of TC transport was motor vehicle accidents (adjusted OR 2.5, 95% CI 1.6–4.0) [58] The rates of falls increased with age (12% of patients aged 16–25 years vs. 77% of patients aged > 65 years) whereas the rates of motor vehicle collisions increased with age (52% of patients aged 16–25 years vs. 16% of patients aged > 65 years) [49] Most of injuries among older adults aged ≥ 65 years occurred at home usually due to falls from standing height (62%) which was the most common mechanism of injury among this population [58]. Falls from standing was shown to decrease the odds of TC transport by 53% (adjusted OR 0.47, 95% CI 0.33–0.67) [58] Most (63%) of older trauma patients aged > 55 who had falls were transported to non-TC [53] Another nationwide research in the USA showed that 71.3% of older trauma patients who were transported to non-TC had falls as their mechanism of injury [42] The sensitivity of prehospital trauma triage criteria, according to the mechanism of injury, is significantly poor to falls (94% under-triage) [39] Falls has a significant impact on the population of injured older adults as it is responsible for 70% of their hospitalisation and 45% of the resulted major trauma among this population [39]
***Other factors not related to trauma triage criteria affecting accurate prehospital triage decisions***	
Socioeconomic factors (sex, age, ethnicity, household income, population density, and geographical location)	For trauma patients aged ≥ 55 years, sex, race, median household income, NISS injury severity, geographic location, mechanism of injury, and number of chronic conditions were statistically significant predictors of TC transport (*p* < 0.0001) except primary payer (*p* = 0.099) [41] The socioeconomic factors identified as predictors of TC transport for trauma patients aged ≥ 55 years included Asian/Pacific and Hispanic race/ethnicity (OR 2.51, CI 1.92–3.27; OR 1.1, CI 0.86–1.42), highest median household income (OR 1.24, CI 1.01–1.52), and high population density (OR 1.32, CI 1.12–1.55; OR 3.2, CI 2.68–2.83) [41] The socioeconomic factors identified as predictors of non-TC transport for trauma patients aged ≥ 55 years included older age groups (OR 0.92, CI 0.76–1.11; OR 0.79, CI 0.64–0.96; OR 0.77, CI 0.63–0.95), females (OR 0.65, CI 0.57–0.74), Black and "other" race (OR 0.75, CI 0.0.56–1.0; OR 0.96, CI 0.77–1.20), lower median household income (OR 0.76, CI 0.62–0.93; OR 0.86, CI 0.71–1.05), low population density (OR 0.96, CI 0.67–1.36; OR 0.89, CI 0.53–1.51), and number of chronic conditions (OR 0.89, CI 0.87–0.91); indicating a risk of bias which needs further assessment and investigation [41] For trauma patients aged > 65 years, higher under-triage rates were identified in rural areas which have limited access to a TC [56]. The regions with low under-triage rates tended to have higher population density compared to regions with middle or high under-triage rates. It also, at the patient level, had more racial and ethnic diversity, higher injury severity, high rates of treatment at a TC [56] For trauma patients aged ≥ 65 years, female patients had increased odds of under-triage (OR 1.09; 95% CI 1.07–1.11). Hispanic patients (OR 1.33; 95% CI 1.25–1.41) and Asian patients (OR 1.28; 95% CI 1.21–1.35) also had higher odds of under-triage compared with white patients [57] Evidence from Australia also showed that one of the positive predictors of TC transport was the male sex (adjusted OR 1.4, 95% CI 1.1–1.8) [58] Among trauma patients aged ≥ 55 years, those who were treated at non-TCs were older (i.e. ≥ 65 year) compared to the patients treated at TCs (82% vs 64%) [55]
Patient or relative choice	Seventy-three percent of hospital selections for older trauma patients were driven by patient or relative choice, however, there were inconsistent findings regarding the benefits this may confer in terms of improving care for older patients [52]
Paramedic-related factors (training, familiarity with protocols, possible ageism, and feeling unwelcomed)	Surveys of prehospital personnel have shown that insufficient training in the management of injured older victims (20%), lack of familiarity with protocols (10%), age bias (such as feeling older people are not worth the extra expenditure [5%] and poor prognoses [2%]), and feeling unwelcomed when bringing patients to a TC (2%) are other possible factors explaining destination non-compliance for older patients who meet the triage criteria [46]

### Developing specific trauma triage criteria

Some scholars have argued that all trauma systems and centres should have specific and effective guidelines for triaging and managing older trauma patients [49]. This has led to further research focusing on the development of specific trauma triage guidelines for older people. Some studies modified the current trauma triage guidelines while others developed separate and specific triage criteria for older trauma patients.

Applying age (age boundary of > 55 years) as a mandatory triage criterion in the trauma triage tool was shown to significantly increase the rates of over-triage as one additional patient with severe injury (ISS > 15) was identified for every 60–65 patients with less severe injuries to be transported to TCs [51]. However, applying the Systolic Blood Pressure (SBP) < 110 mm Hg instead of the SBP < 90 mm Hg for triaging older trauma patients in prehospital care decreased the under-triage rate by 4% and increased the over-triage rate by 4% [36]. As the risk of death for older trauma patients who had a SBP < 110 mm Hg is similar to those who had a SBP < 90 mm Hg, this finding may require the application of this criterion for the direct transportation of these patients to TCs [36]. Furthermore, for trauma patients aged ≥ 70 years, applying the criterion of Glasgow Coma Scale (GCS) ≤ 14 instead GCS ≤ 13 increased the sensitivity rate of the triage tool in this population from 50.7 (95% CI 47.5–53.9) to 59.2% (95% CI 56.1–62.3) with a similar specificity rate to the criterion GCS ≤ 13 in younger adults (85.1% [95% CI 84.6–85.7] vs. 85% [95% CI 84.6–85.4], respectively) [54].

Some other studies developed separate and specific trauma triage criteria for older adults. For example, Newgard et al. [52] developed an alternative trauma triage tool for trauma patients aged ≥ 65 years that, compared to current adult trauma triage guidelines, showed better sensitivity in identifying major trauma (ISS > 15) (92% vs. 76%) but significantly less specificity (42% vs. 78%). Furthermore, Newgard and colleagues [43] developed a clinical decision rule for triaging older trauma patients which aligned the current triage criteria with geriatric-specific physiology and comorbidity criteria. The overall sensitivity of this decision rule was 90%, however, it had significantly low specificity (17%) to identify injured older adults who had an ISS > 15 or those who require major non-orthopaedic surgery. Adding the use of anticoagulants in the decision rule was not shown to be a good predictor of high-risk patients when compared to current triage criteria, geriatric-specific physiologic measures, and comorbidities [43]. However, the geriatric-specific trauma triage guidelines developed by the Trauma Committee of the Ohio Emergency Medical Services Board showed increased sensitivity for patients aged ≥ 70 years compared to current adult trauma triage criteria (93% vs. 61%) and decreased specificity (49% vs. 61%) [48]. Furthermore, the performance of this developed triage tool in geriatric trauma patients was similar to the performance of the current triage tool in younger trauma patients (sensitivity: 93% vs. 87%, and specificity: 49% vs. 44%, respectively) [48]. Nevertheless, using the cut-off point of ≥ 70 years represents a major limitation in this triage tool as a previous study has shown that under-triage issues can begin as early as the age of 50 years [46].

### Trauma triage destination compliance

‘Destination compliance’ is defined as access to the highest level of trauma services for patients who meet the prehospital trauma triage criteria. An example of this is that if a trauma patient with a GCS 8 was assessed by a paramedic to have severe injury and met the triage criteria for TC transport, he/she was actually transported to a TC. In this section, we are going to discuss the rates of destination compliance and the possible contributing factors leading to destination non-compliance for older trauma patients meeting the prehospital triage criteria.

#### Destination compliance and current triage criteria

For injured patients meeting current triage criteria, there was a constant decrease in the rate of destination compliance with increasing age [37, 50, 51]. Trauma patients aged > 60 years had a greater under-triage rate when defined by hospital destination; indicating that older trauma patients meeting the triage criteria were not as likely to be transported to TCs [51]. In Australia, 67% of older confirmed major trauma patients who met the triage criteria were transported to a TC in comparison with 88% of younger patients [37]. In the USA, older trauma patients, when they met the triage criteria, were only half as likely as younger adults to be transported to designated TCs [46]. More recent evidence from the USA also showed that of all older trauma patients meeting the triage criteria, only 38% were transported to level I/II TCs [43]. Significantly high rates of under-triage was identified in older trauma patients whose closest facility was non-TC whether it had less than 200 beds or had 200 or more beds (adjusted OR 4.48, *P* < 0.001; adjusted OR 8.53, *P* < 0.001, respectively) [56].

For older trauma patients meeting the physiological criteria of the prehospital triage tool, another piece of more recent evidence showed that only 24% of hypotensive patients (SBP < 90 mmHg), 22.6% of those who had an abnormal respiratory rate (< 10 or > 29 breaths per minute), and 26% of those who had a GCS < 13 were transported to a TC [50]. With respect to distance, even when older patients were injured in places close to a TC, they were less likely to be transported to these centres than younger adults [47]. Indeed, 55.8% of older trauma patients living in urban regions were transported to lower or non-TCs [42].

#### Destination compliance and developed geriatric-specific triage criteria

Caterino et al. [45] found that developing geriatric-specific triage criteria significantly improved the identification of trauma, however, the rate of initial transportation only increased by 1% while the rate of initial transportation and inter-hospital transfers increased by 2%; indicating an issue of destination non-compliance for patients meeting the triage criteria. In the USA, only 47% of older patients who met the geriatric-specific trauma triage criteria were initially transported to level I/II TCs and 59% were ultimately transported to these centres [44]. Of those, patients who lived in regions with level I/II TCs had the highest rates of being transported to these centres whereas patients in regions with level III TCs had the lowest rates of higher-level TC transport [44]. This means that patients in regions with no TCs had a better chance for level I or II TC transport either initially or ultimately than those in regions with level III TCs [44].

#### Factors leading to destination non-compliance

It is still unclear which factors have led to under-triage among older trauma patients; reduced sensitivity of the triage guidelines or factors such as ageism, patient choice, or other inherent variations relevant to indications of severe trauma in this population [51]. It is also argued that decision-making during the triage of older trauma patients could be affected by paramedics’ subjective judgments [37].

One of the possible factors leading to destination noncompliance for older trauma patients is patient or relative choice as evidence showed that most hospital selections were driven by the choice of the patient or relative [52] (Table 4). However, there were inconsistent findings regarding the benefits this may confer in terms of improving care for older patients [52]. Furthermore, the assessment of socioeconomic factors showed a possibility of bias with respect to ethnicity, age, and sex (towards female sex) which needs more rigorous assessment and investigation [41, 55–57] (Table 4). Additionally, surveys of paramedics showed other paramedic-related factors which could impact appropriate triage decisions for older trauma patients including insufficient training, unfamiliarity with protocols, possible ageism, and feeling unwelcomed when bringing patients to a TC [46] (Table 4).

### Trauma triage and outcomes

Older patients had significantly higher mortality than younger adults following injury [37, 47]. The odds of death for trauma patients increased by 8% for each year at the age cut-off > 55 years (OR 1.08; 95% CI 1.07–1.09) [37]. This is consistent with other research findings which found increasing mortality rates with age [39, 40]; highlighting the need for a better understanding of trauma outcomes for older patients. Larger proportion of older patients died at non-TCs (32%) than TCs (20%) [37]. The lower odds of transporting older trauma patients to TCs was associated with 1.7 times increased likelihood of their in-hospital deaths (95% CI 1.04–2.7) [58]. Under-triaged older trauma patients, compared to younger adults, had higher rates of mortality (21% vs. 6%), disability (22% vs. 6%), and complications (39% vs. 23%) [49].

#### Benefits of trauma centre access

A recent study by Garwe et al. [55] found contradicting evidence as the TC treatment for patients aged ≥ 55 years was associated with longer in-hospital stay (mean [SD], 7.6 (7.2) vs 5.8 (5.6), *p* < 0.0001) and had higher un-adjusted mortality (10.2% vs. 7.5%, *p* < 0.0001) than those treated at non-TCs [55]. In the multivariate Cox regression analyses, treatment at TCs was significantly associated with a lower likelihood of death within the first 7-days after adjusting for potential confounding factors and this effect was much stronger for patients aged 55 to 64 years (Hazard Ratio [HR] 0.45, 95% CI 0.36–0.56) compared to those aged ≥ 65 years (HR 0.65, 95% CI 0.58–0.73) [55]. The protective effect for treating patients at TCs was also observed in those who survived beyond 7 days (HR 0.69, 95% CI 0.56–0.83) [55]. Furthermore, after adjusting for potential confounders, transfer to TCs was associated with significantly lower 30-day mortality for patients aged 55 to 64 years (HR 0.36, 95% CI 0.27–0.49) and also for those aged ≥ 65 years (HR 0.55, 95% CI 0.48–0.64) [55]. Another piece of evidence also showed that the treatment of trauma patients aged ≥ 65 years at TCs was associated with a significant positive change in the probability of survival (marginal effect of 3.9% at the 5% level) although, when patients were stratified by age, patients aged ≥ 85 years had no statistical significance of the effect of TC treatment on their probability of survival at the 5% level (marginal effect of 3.6%) compared to patients aged 65 to 74 years (marginal effect of 7%) and patients aged 75 to 84 years (marginal effect of 4%) [40]. Conversely, Staudenmayer et al*.* [53] showed that the unadjusted 60-day mortality rate for older trauma patients transported to non-TC with an ISS > 15 was not significantly different from those with the same score who were transported to TCs (16% vs. 17%, p = 0.87). The transportation of older patients with major trauma (ISS > 15) to TCs was associated with higher costs than non-TCs (Total costs, $, median [IQR], 35,069 [19,321—88,357] vs. 14,332 [5112—29,321]) and longer in-hospital stays (Length of stay, days, median [IQR], 6.0 [3.0—14.0] vs. 5.0 [1.5—8.0]) [53]. However, the study did not adjust for important confounders such as comorbidities, specific patterns of injury such as head injury, and the mortality analysis was based on only 41 deaths which may have impacted the precision of the estimated effect [53].

#### Outcomes and the development of geriatric-specific trauma triage criteria

The application of a geriatric-specific trauma triage tool was shown to have no significant difference in mortality rates after its application compared to pre-application (7%), which may be attributed to destination non-compliance for patients meeting the developed triage criteria [45]. Patients with ISS < 10 were the only group who benefit from applying the developed triage tool (decreased mortality from 3.0 to 2.5%) [45]. Evidence showed that a large proportion of older trauma patients died after sustaining minor to mild injuries [53]. In contrast, ISS > 15 was shown to be the highest level of serious injury that predicts increased in-hospital mortality rates for injured patients aged ≥ 65 [52]. Applying the geriatric-specific trauma triage tool resulted in a minimal unadjusted increase from 34% (95% CI 33–35%) to 35% (95% CI 35–35%) in the number of older patients discharged home (difference 1.2%, 95% CI of the difference 0.2–2.2%) (*p* = 0.02) [45].

For trauma patients aged ≥ 70 years, a decline in the GCS from 15 to 14 was associated with increased mortality (OR 1.40; 95% CI 1.07–1.83), which was not the case for younger adults (OR 1.22; 95% CI 0.88–1.71). Similarly, a decline in the GCS from 14 to 13 was also associated with increased mortality for older patients (OR 2.34; 95% CI 1.57–3.52) but not for younger adults (OR 1.45; 95% CI 0.91–2.30) [54]. Moreover, patients aged ≥ 70 years with a GCS of 14 were shown to have higher odds of mortality (OR 4.68; 95% CI 2.90–7.54) and traumatic brain injuries (OR 1.84; 95% CI 1.45–2.34) than younger adults with GCS 13; suggesting the need for modifying the GCS criterion for older trauma patients so they can possibly get the advantage of direct transport to TCs [54].

## Discussion

This review showed that the findings from relevant literature showed significant rates of under-triage for older trauma patients in prehospital care. There was no substantial qualitative difference in the older versus more recent studies. Most of the current and developed trauma triage guidelines lack acceptable accuracy to identify major trauma or were not applied to all older adults. Lower rates of destination compliance represent a major issue for this population to access TCs. The review showed that the association between under-triage of seriously injured older patients and high mortality rates is inconclusive. There was a conflict of evidence about the benefits of TC access for older trauma patients with regards to their survival or mortality rates. Some papers found that under-triage is associated with high rates of mortality, disability, and complications for older trauma patients. Treatment at TCs was associated with significantly higher costs and a lengthier in-hospital stay than at non-TCs.

Older trauma patients usually have injuries compounded with multimorbidity and frailty. This could adversely affect the accuracy of prehospital trauma triage tools even when geriatric-specific triage criteria were developed to adjust for age-related anatomical and physiological changes, comorbidities, and medication use [61], as shown in this review. Therefore, integrating other assessment tools, such as frailty, into the trauma triage tool may improve the identification of high-risk patients and reduce under-triage [62]. However, paramedics’ compliance to trauma triage tools was shown to decrease with patients aged ≥ 55 years compared to younger adults [63], which is consistent with our findings. The compliance rate of paramedics to trauma triage tools ranged from 21 to 93% [63]; affecting the usefulness of applying frailty-attuned scores in prehospital care.

Understanding research priorities in geriatric trauma is complex including prehospital care [64]. Quick and accurate prehospital triage and transportation decisions for older trauma patients are needed and this review has shown that the majority of the recent efforts in the literature are looking into this area intensively. However, a recent consensus-building exercise determined that assessing the benefits of TC access for older trauma patients to be more important [65]. Our review showed that some literature found some survival benefit of triaging and transporting older trauma patients to TCs, but these findings are still uncertain. The treatment at TCs was, however, shown to increase the length of in-hospital stay and costs. These findings were consistent with other research findings which showed an inconclusive survival benefit of TC access for this population [62].

Most studies looking into outcomes in this review assessed the impact of prehospital triage decisions mainly on mortality or survival rates. No standardised Patient-Reported Outcome Measures (PROMs) were used to assess patient outcomes. A recent review highlighted the importance of assessing outcomes beyond mortality for older trauma patients [62]. It also argued that assessing both clinical and patient outcomes are important for this population to strike an ethical balance between paternalism (in healthcare provision) and autonomy (of the patient in what they expect and seek) when caring for this population [62]. Therefore, it is important to determine appropriate Clinician-Reported Outcome Measures (CROMs) and PROMs to appropriately assess the impact of prehospital trauma triage decisions and the benefits TC care for this population.

This systematic review is the first review to assess prehospital triage specifically for older trauma patients using a thematic approach. The review followed a systematic approach in searching the literature, quality assessment, and presenting the findings which was performed independently by two reviewers. However, there were some limitations evident in this review that should be highlighted. The review was restricted to papers published in English which could preclude the results from non-English papers that may have impacted the findings. Furthermore, due to the heterogeneity of the included studies, we were unable to perform a meta-analysis and a sub-group comparison between older and more recent studies.

The review has several significant implications. Because all the studies followed a retrospective design, this precludes the measurement of key factors that may have had a bearing on the findings. More studies utilising a high-quality prospective design are needed to further assess the effectiveness of current trauma triage tools and destination compliance for older patients. Furthermore, there is a need to develop more accurate geriatric-specific triage criteria and assess the integration of frailty assessment into the triage tool. The impact of triaging older trauma patients to TCs should be assessed more on both clinical and patient outcomes. Moreover, studies focusing on different healthcare systems in other countries are required to further enhance the application of effective prehospital trauma triage for older patients.

## Conclusion

Prehospital under-triage of older trauma patients seems to be an ongoing issue that requires future high-quality prospective research to assess and improve the accuracy of prehospital triage criteria for this population. Decreased compliance with prehospital triage decisions for positively triaged patients with age was identified in this review and the factors leading to this issue needs further investigation and assessment. The impact of this issue on the outcomes is still uncertain. There are no standardised outcome measures for older trauma patients that can be reliably measured to assess the impact of prehospital triage decisions.

## Supplementary Information

Below is the link to the electronic supplementary material.

Supplementary file1 (DOCX 31 kb)

## Abbreviations

RR: Relative Risk.
TTDP: Trauma Triage Destination Plans
AUC: Area Under the Curve
PROMs: Patient-Reported Outcome Measures
CROMs: Clinician-Reported Outcome Measures
AIS: Abbreviated Injury Scale
CASP: Critical Appraisal Skills Programme
UK: United Kingdom
USA: United States of Americ
ISS: Injury Severity Score
TC: Trauma Centre
OR: Odds Ratio
CI: Confidence Interval
PICO: Population, Intervention, Comparison, and Outcome
PRISMA: Preferred Reporting Items for Systematic Reviews and Meta-Analyses
NISS: New Injury Severity Score
HEMS: Helicopter Emergency Medical Services
FTTA: Florida Trauma Triage Algorithm
IQR: Interquartile Range
SBP: Systolic Blood Pressure
HR: Heart Rate
GCS: Glasgow Coma Scale
SD: Standard Deviation
HR: Hazard Ratio
