# Supplementary material for: Under-triage of older trauma patients in prehospital care: a systematic review
Source: Eur Geriatr Med. 2021 Jun 10;12(5):903–19. doi: 10.1007/s41999-021-00512-5 (PMC8463357; doi:10.1007/s41999-021-00512-5)
Supplement: Supplementary file 1 — Supplementary file1 (DOCX 31 kb) [file 41999_2021_512_MOESM1_ESM.docx]

| Study | Themes | | | | |
| --- | --- | --- | --- | --- | --- |
|  | Under-triage rates. | Clinical effectiveness of current trauma triage criteria. | Developing specific trauma triage criteria. | Trauma triage and destination compliance. | Trauma triage and outcomes. |
| Amoako et al. (2019) [44] | 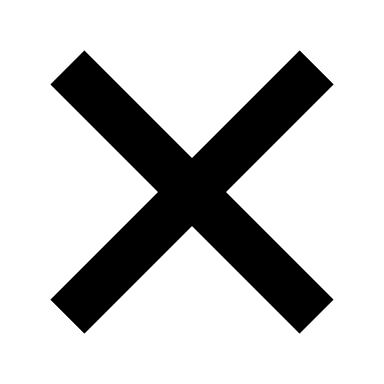 | 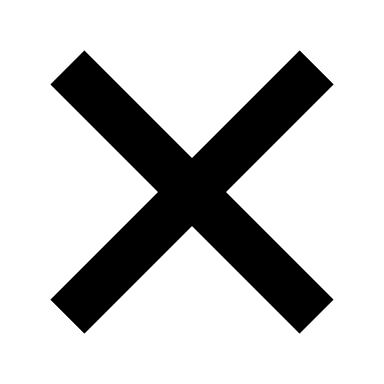 | 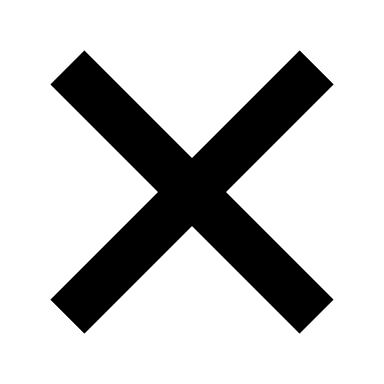 | 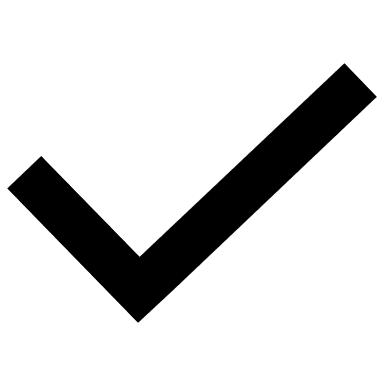 | 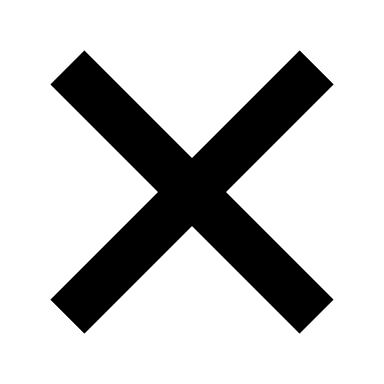 |
| Brown et al. (2019) [58] | 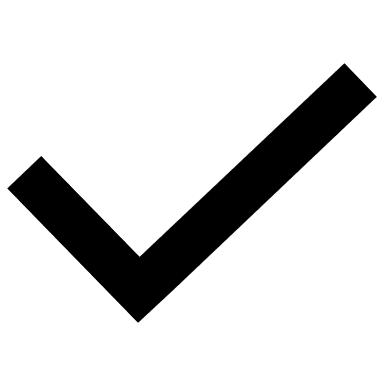 | 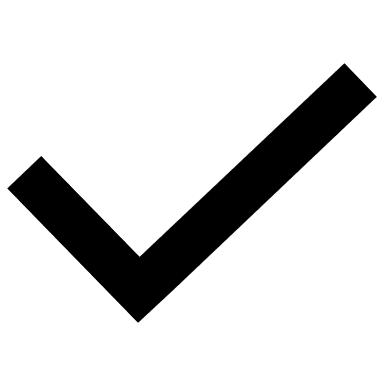 | 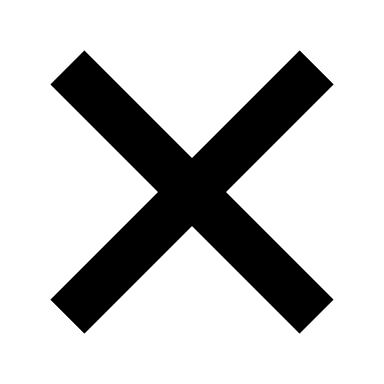 | 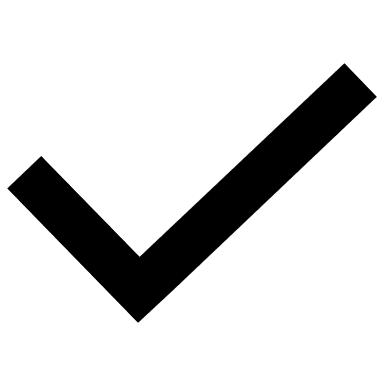 | 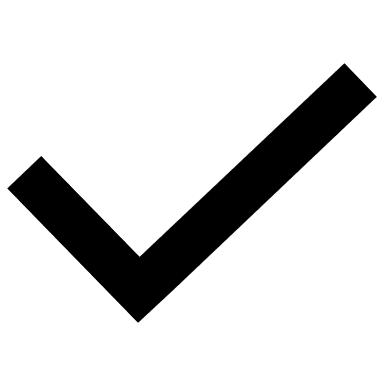 |
| Brown et al. (2015) [36] | 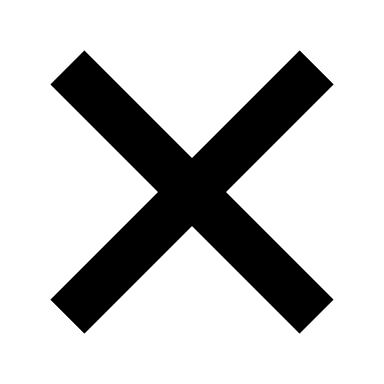 | 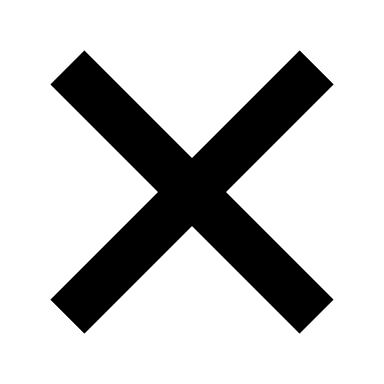 | 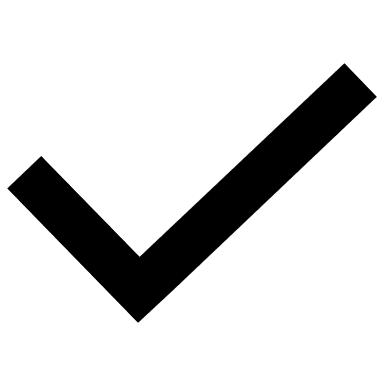 | 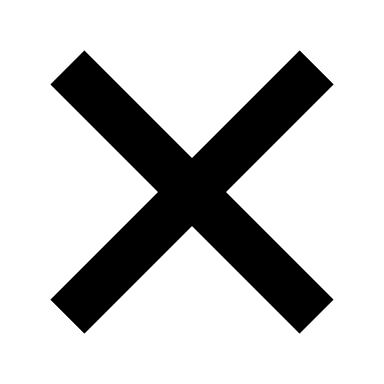 | 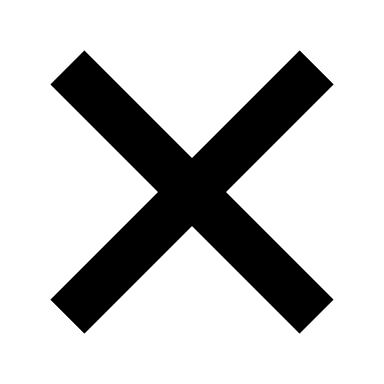 |
| Caterino et al. (2016) [45] | 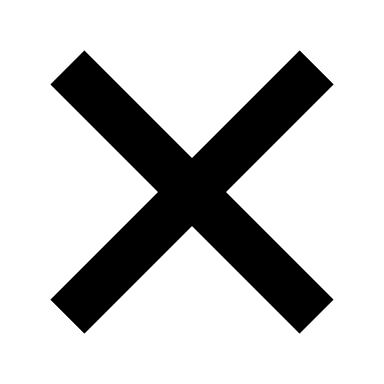 | 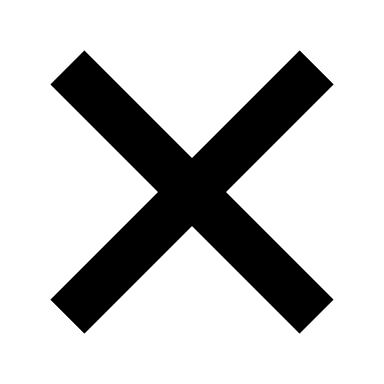 | 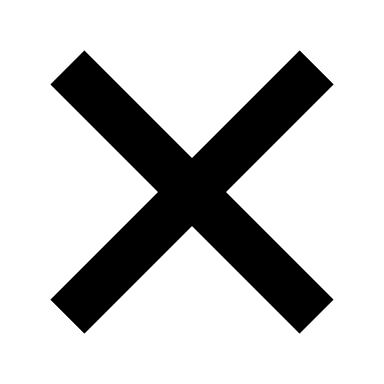 | 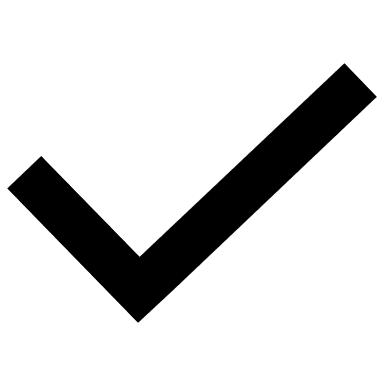 | 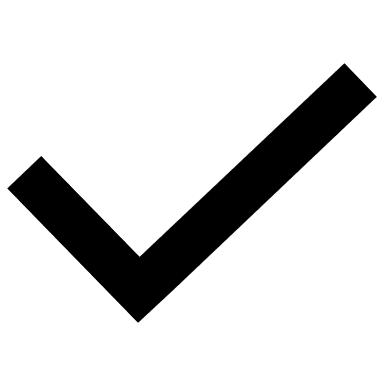 |
| Caterino et al. (2011) [54] | 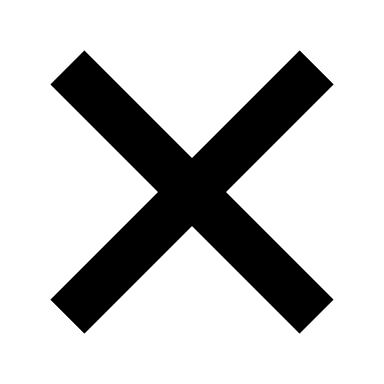 | 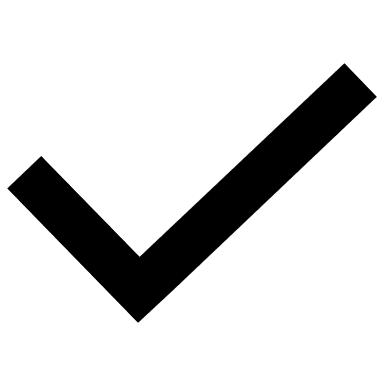 | 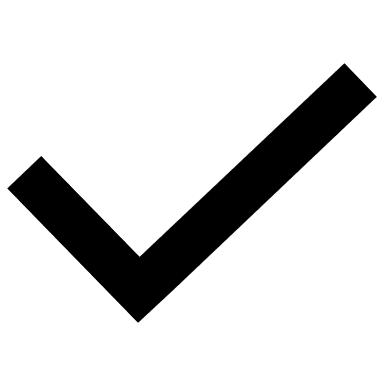 | 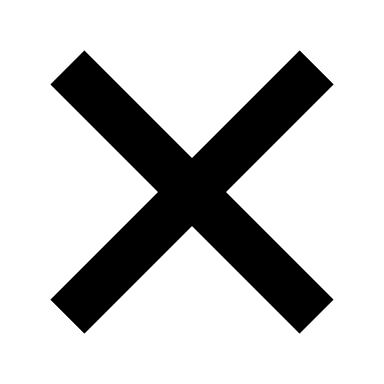 | 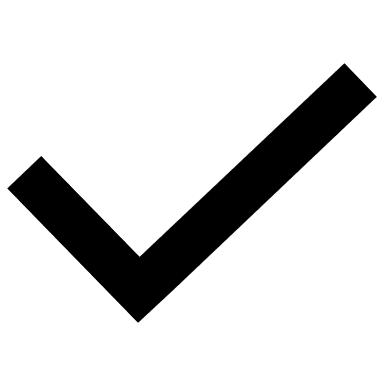 |
| Chang et al. (2008) [46] | 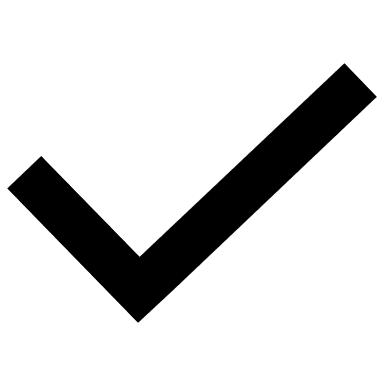 | 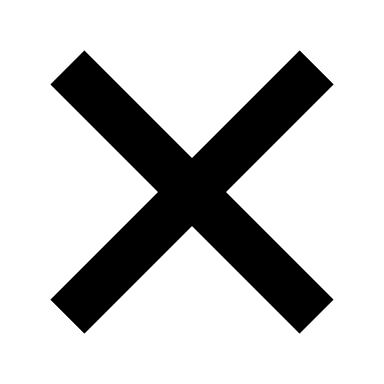 | 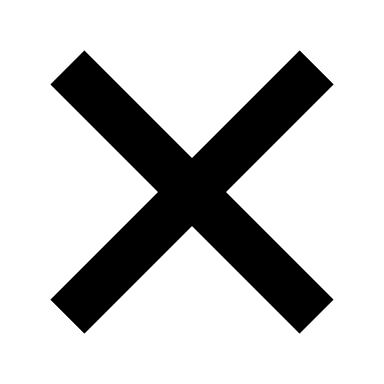 | 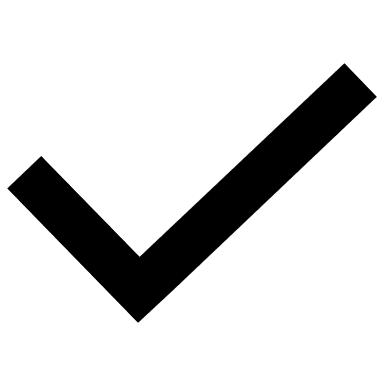 | 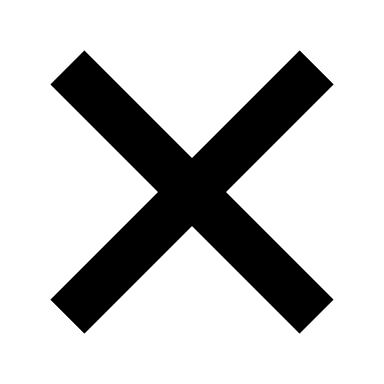 |
| Cox et al. (2014) [37] | 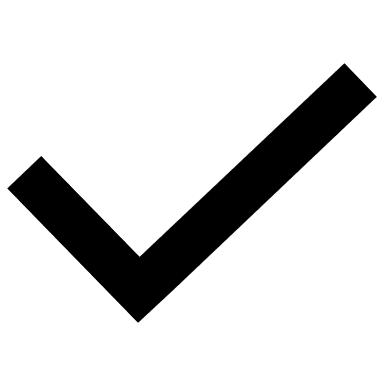 | 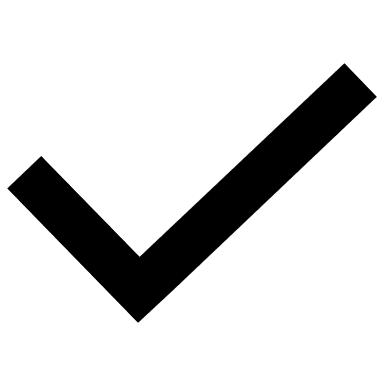 | 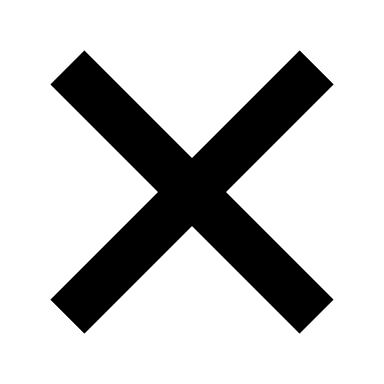 | 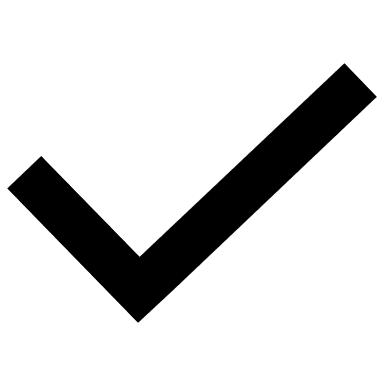 | 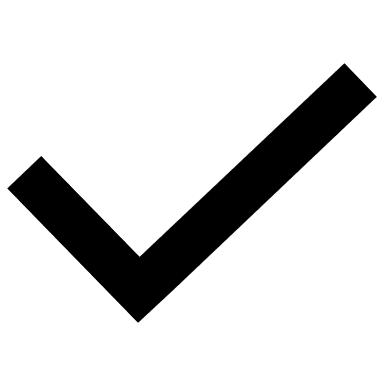 |
| Davis et al. (2012) [38] | 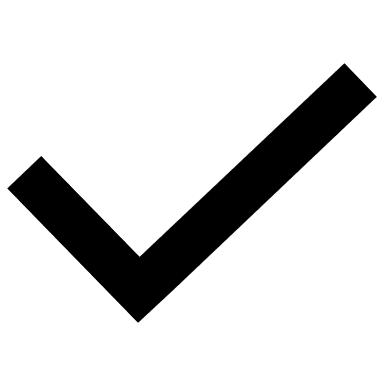 | 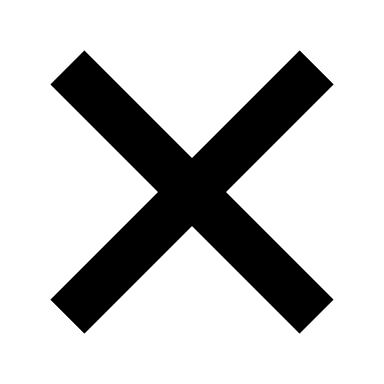 | 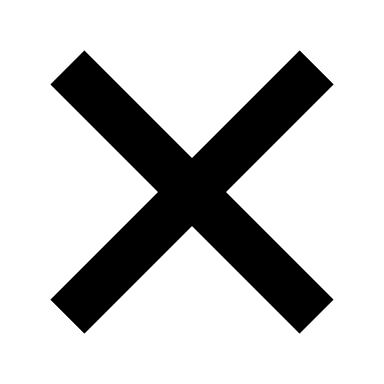 | 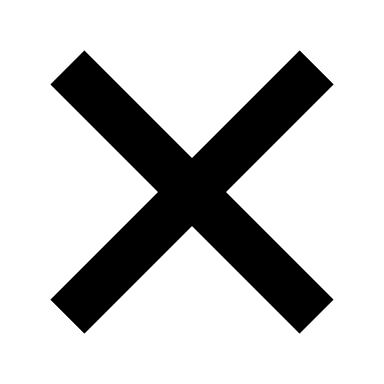 | 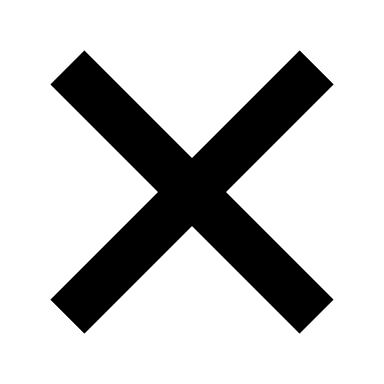 |
| Garwe et al. (2017) [47] | 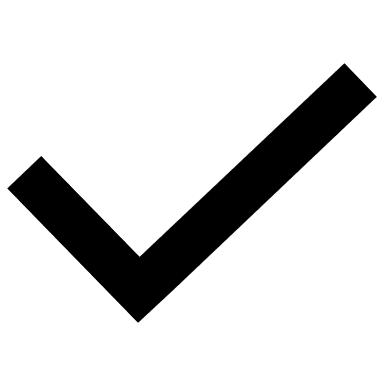 | 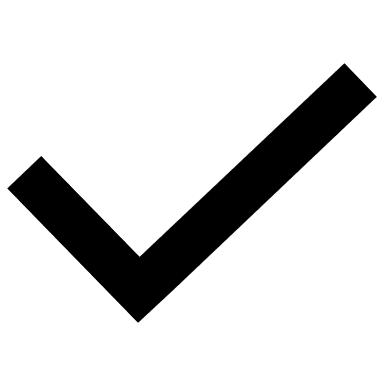 | 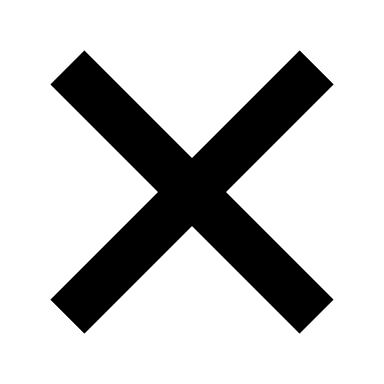 | 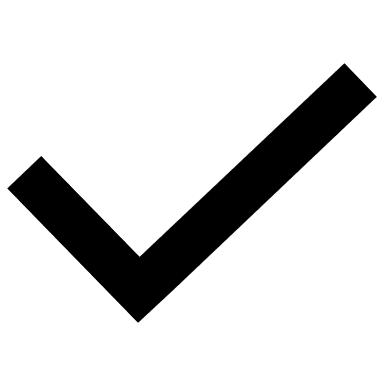 | 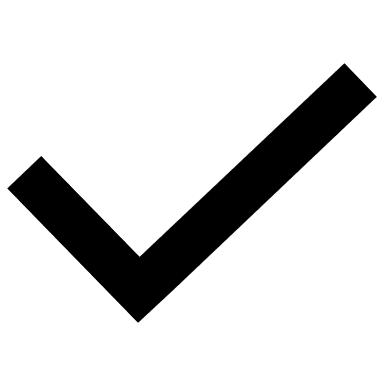 |
| Garwe et al. (2020) [55] | 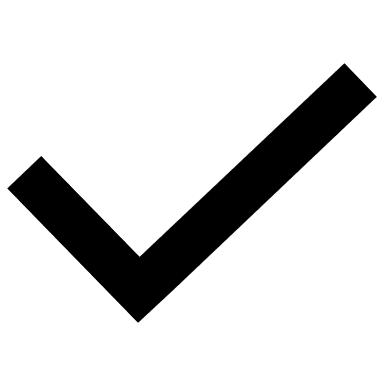 | 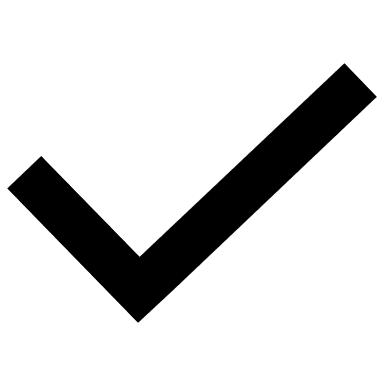 | 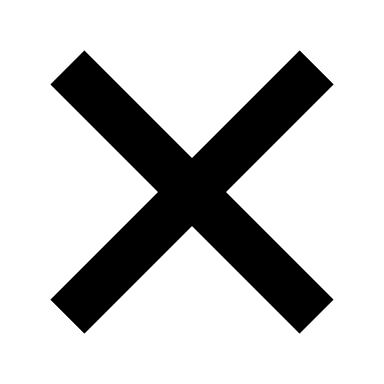 | 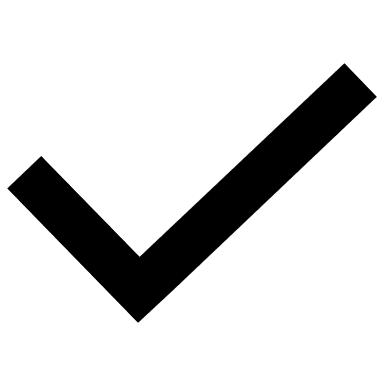 | 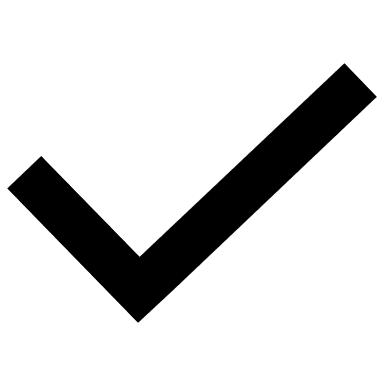 |
| Horst et al. (2020) [56] | 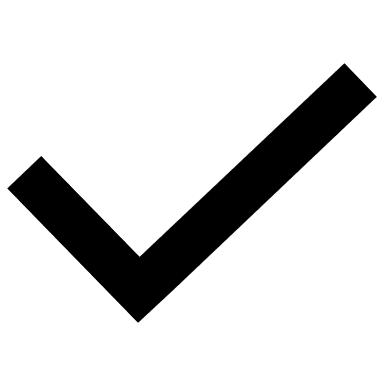 | 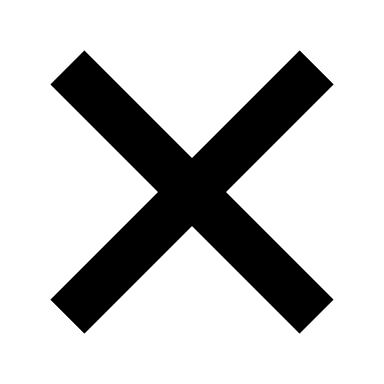 | 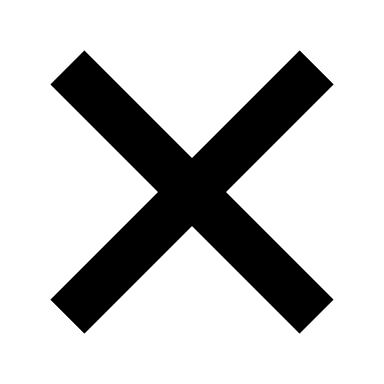 | 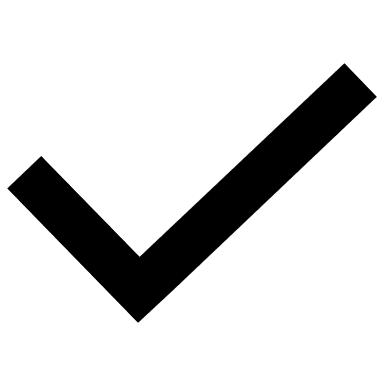 | 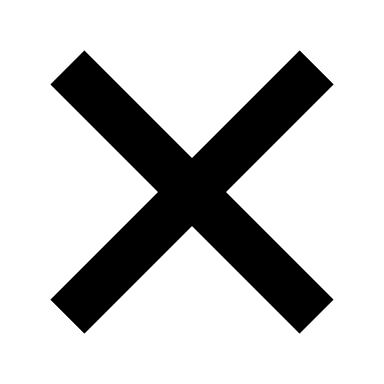 |
| Ichwan et al. (2015) [48] | 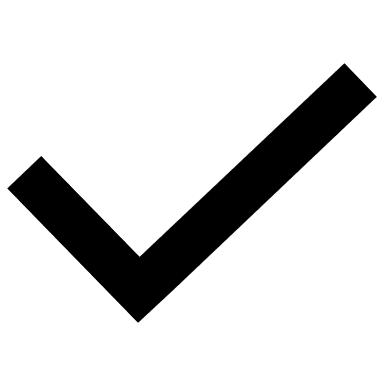 | 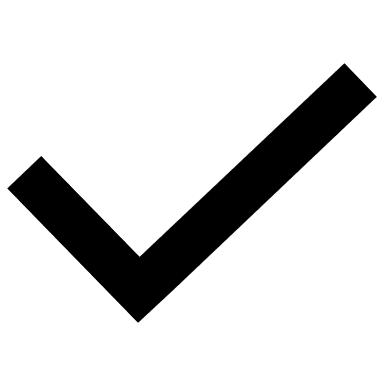 | 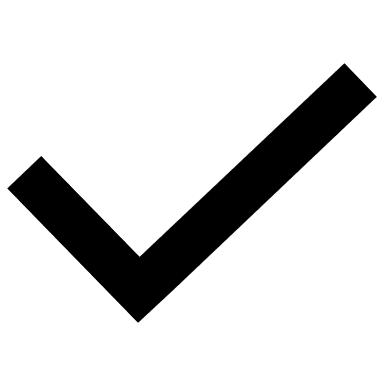 | 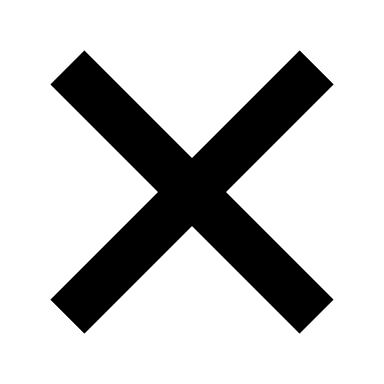 | 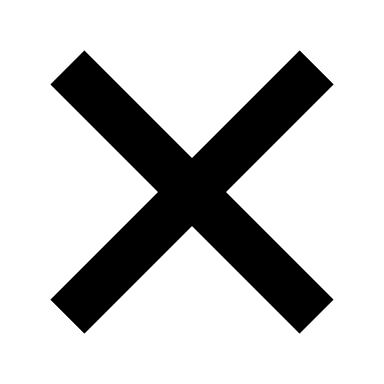 |
| Kodadek et al. (2015) [42] | 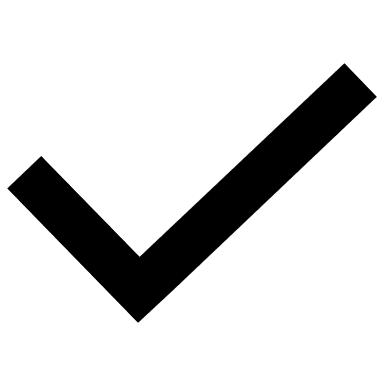 | 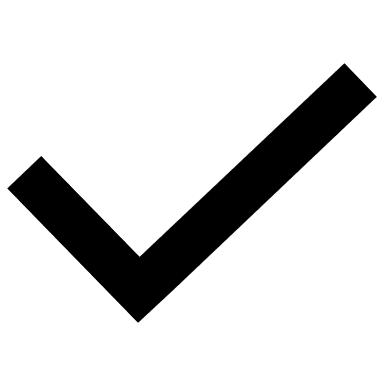 | 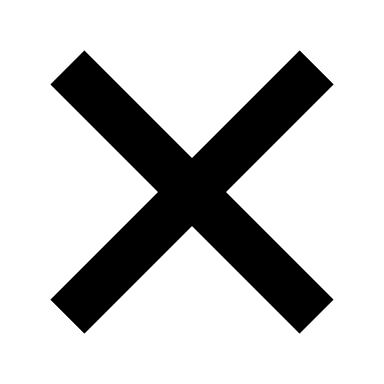 | 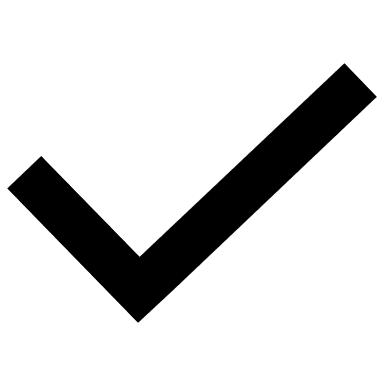 | 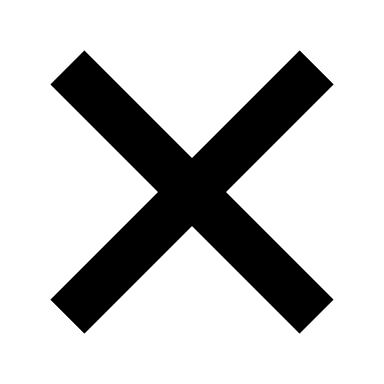 |
| Lehmann et al. (2009) [49] | 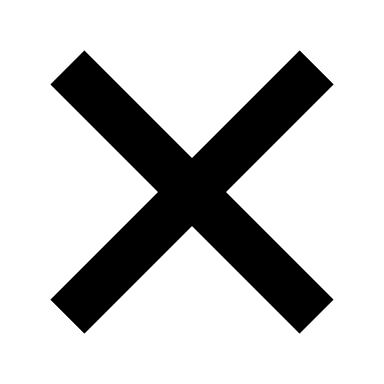 | 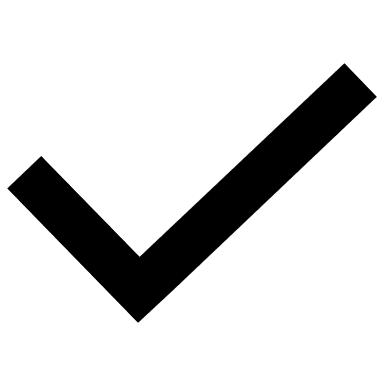 | 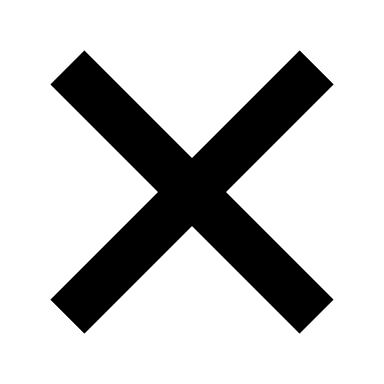 | 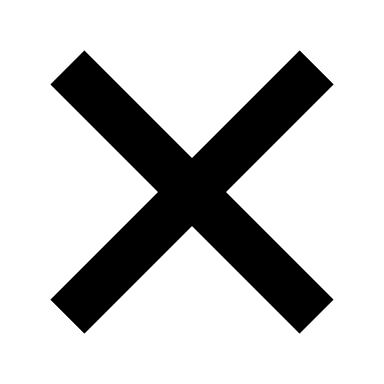 | 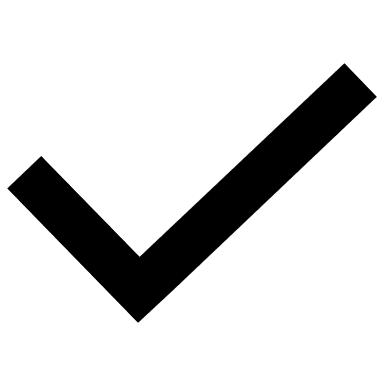 |
| Meyers et al. (2019) [50] | 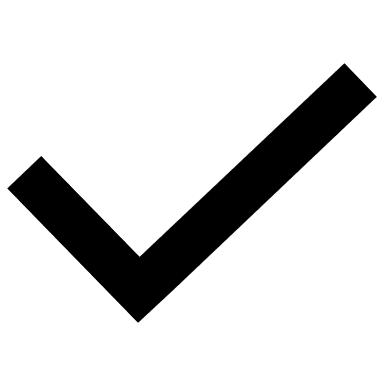 | 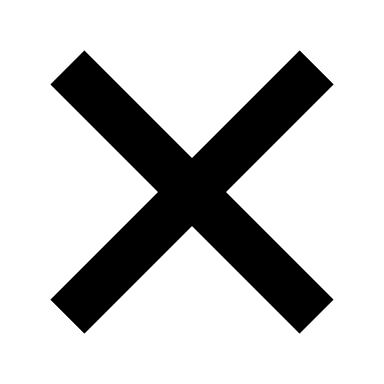 | 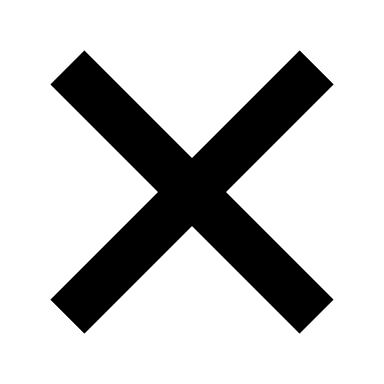 | 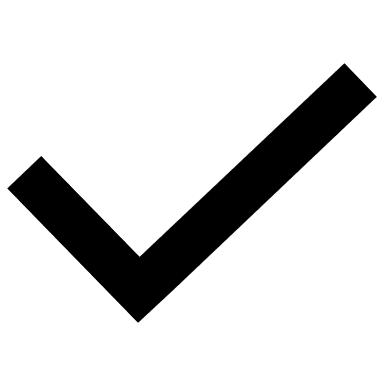 | 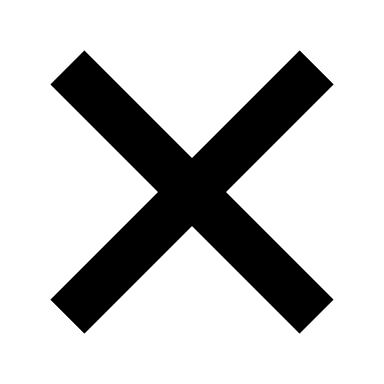 |
| Nakamura et al. (2012) [51] | 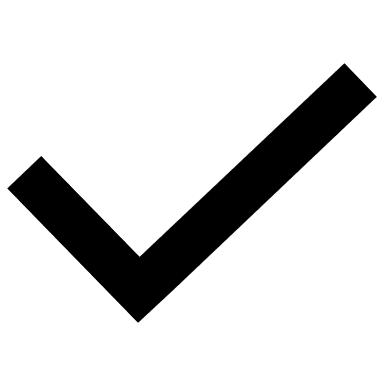 | 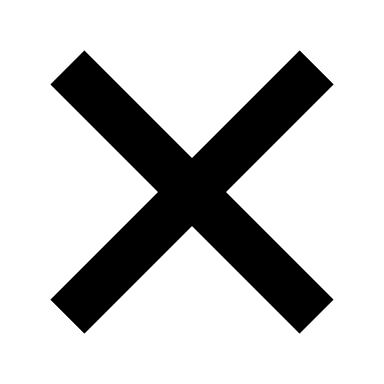 | 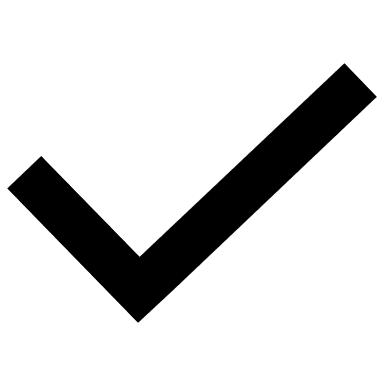 | 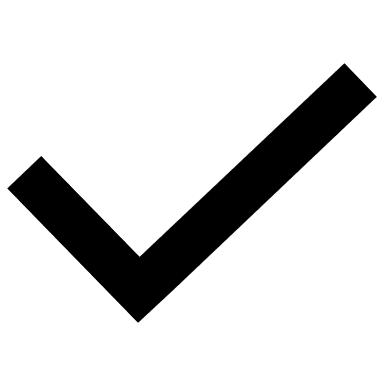 | 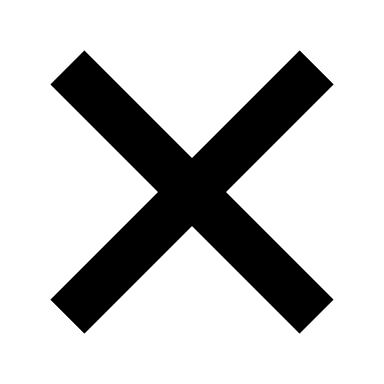 |
| Newgard et al. (2016) [52] | 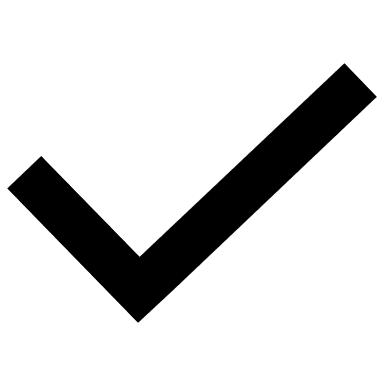 | 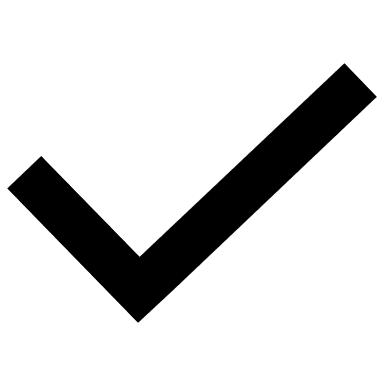 | 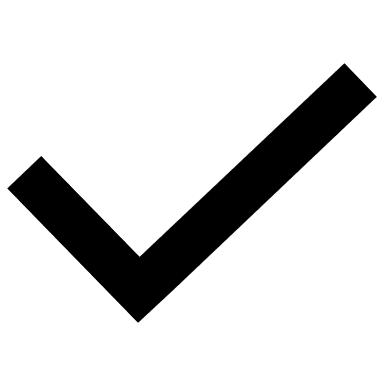 | 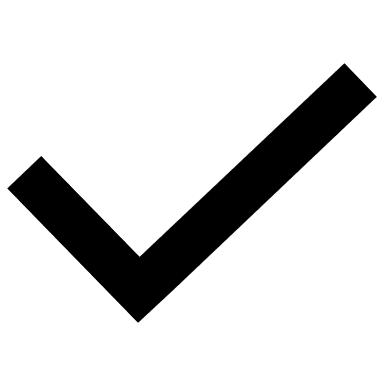 | 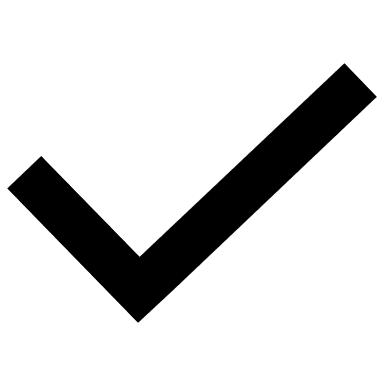 |
| Newgard et al. (2019) [43] | 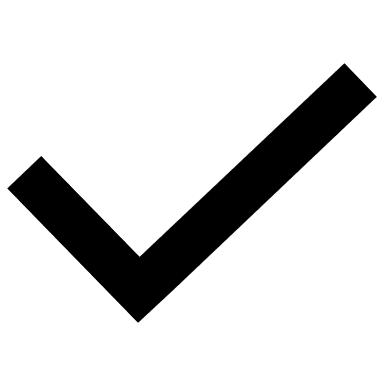 | 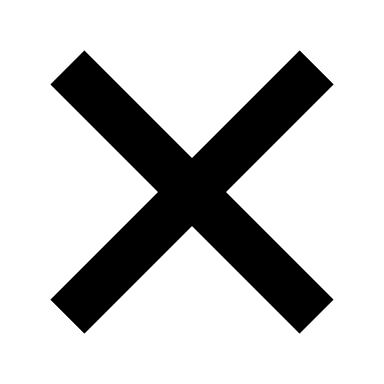 | 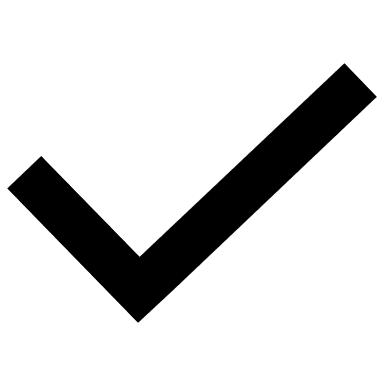 | 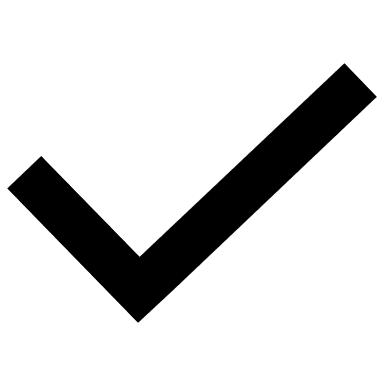 | 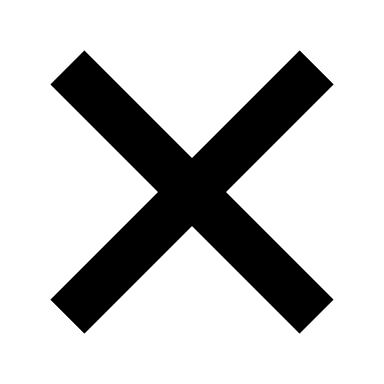 |
| Phillips et al. (1996) [39] | 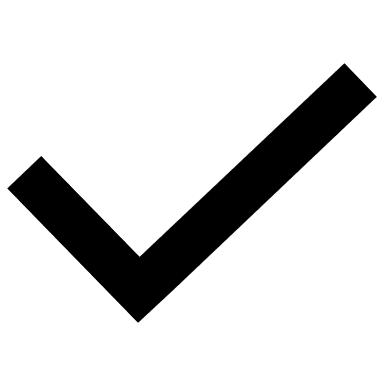 | 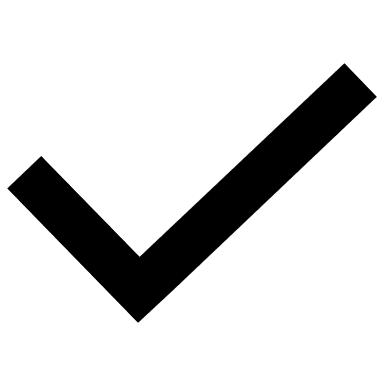 | 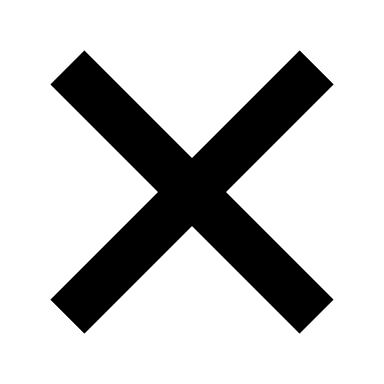 | 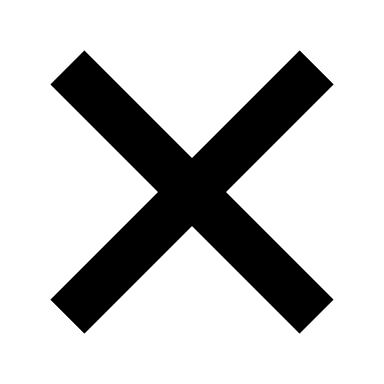 | 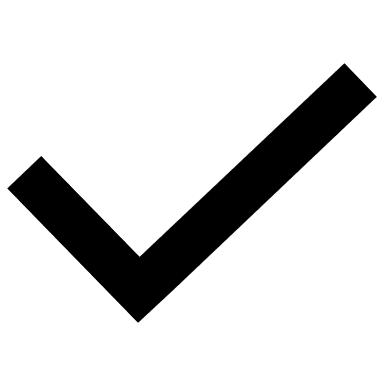 |
| Pracht et al. (2011) [40] | 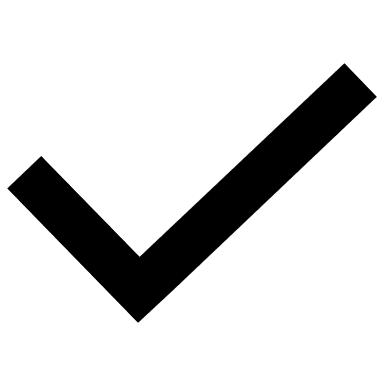 | 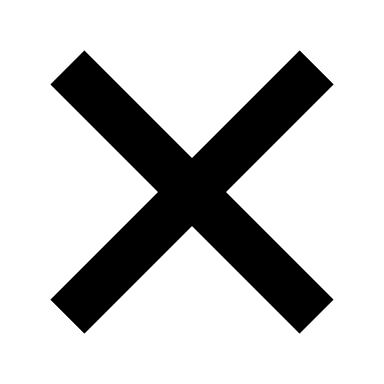 | 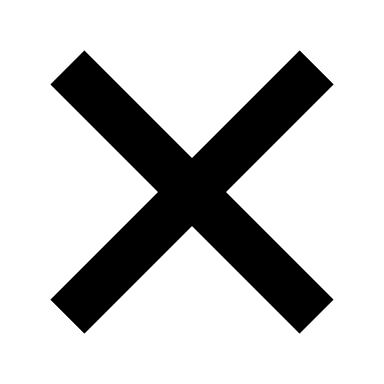 | 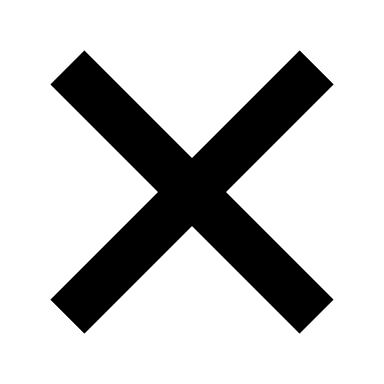 | 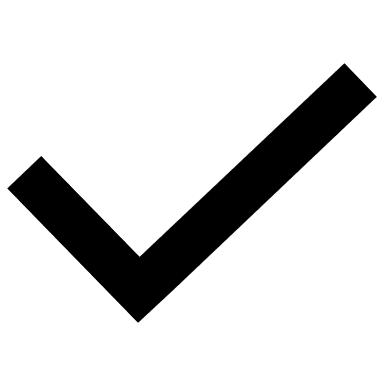 |
| Scheetz and Orazem (2020) [41] |  |  |  |  |  |
| Staudenmayer et al. (2013) [53] |  |  |  |  |  |
| Uribe-Leitz et al. (2020) [57] |  |  |  |  |  |
